# Functional conservation and diversification of the soybean maturity gene *E1* and its homologs in legumes

**DOI:** 10.1038/srep29548

**Published:** 2016-07-13

**Authors:** Xingzheng Zhang, Hong Zhai, Yaying Wang, Xiaojie Tian, Yupeng Zhang, Hongyan Wu, Shixiang Lü, Guang Yang, Yuqiu Li, Lu Wang, Bo Hu, Qingyun Bu, Zhengjun Xia

**Affiliations:** 1 Key Laboratory of Soybean Molecular Design Breeding, Northeast Institute of Geography and Agroecology, Chinese Academy of Sciences, Harbin 150081, China; 2 University of Chinese Academy of Sciences, No. 19A Yuquan Road, Beijing 100049, China; 3 Information Center of Heilongjiang Academy of Agricultural Sciences, Harbin 150086, China

## Abstract

Gene regulatory networks involved in flowering time and photoperiodic responses in legumes remain unknown. Although the major maturity gene *E1* has been successfully deciphered in soybean, knowledge on the functional conservation of this gene is limited to a certain extent to *E1* homologs in legumes. The ectopic expression of *Phvul.009G204600* (*PvE1L*), an *E1* homolog from common bean, delayed the onset of flowering in soybean. By contrast, the ectopic expression of *Medtr2g058520* (*MtE1L*) from *Medicago truncatula* did not affect the flowering of soybean. Characterization of the late-flowering *mte1l* mutant indicated that *MtE1L* promoted flowering in *Medicago truncatula*. Moreover, all transgenic *E1*, *PvE1L* and *MtE1L* soybean lines exhibited phenotypic changes in terms of plant height. Transgenic *E1* or *PvE1L* plants were taller than the wild-type, whereas transgenic *MtE1L* plants produced dwarf phenotype with few nodes and short internode. Thus, functional conservation and diversification of *E1* family genes from legumes in the regulation of flowering and plant growth may be associated with lineage specification and genomic duplication.

Soybean [*Glycine max* (L.) Merr], an important source of plant protein and oil, plays a vital role in ensuring food security. Soybean yield generally depends on the production of seeds to be used as crop. Transition from vegetative stage to reproductive stage is critical in seed production in spermophytes, and flowering time is influenced by a series of environmental and endogenous factors. Many genes in the model plant *Arabidopsis thaliana*, participate in several flowering pathways^1^,^2^,^3^, e.g. photoperiod, gibberellin, ambient temperature, vernalization, aging and autonomous pathways. The photoperiod pathway controls flowering time in response to seasonal changes in day length. Light (day-length) in plants is perceived by photoreceptors in leaves, and downstream signals are regulated by the circadian clock. Florigen is induced and transported into the shoot apex to trigger flowering by inducing the expression of floral meristem identity genes. Two photoperiod responsive genes, namely, *CONSTANS* (*CO*) and *FLOWERING LOCUS T* (*FT*), are vital in regulating photoperiodic flowering. *CO* encoding the B-box zinc finger protein^4^ integrates the circadian clock and photoperiod pathways^5^. By contrast, *FT*, which encodes a phosphatidylethanolamine-binding protein, is induced in leaves and transported into the meristem through the phloem^6^. The *CO-FT* module controls flowering across many plant species, such as rice^7^ and barley^8^.

Genetic analysis revealed 10 flowering and maturity loci (*E1* to *E9*, *J*) in soybean^9^,^10^,^11^,^12^,^13^,^14^,^15^,^16^,^17^. *E1*, *E3*, *E4*, and *E7* are strongly responsive to photoperiod^10^,^11^,^12^,^18^. *E1–E4* were cloned using either candidate gene approach or positional cloning. *E2* encodes *GmGIa*, which is an ortholog of *GIGANTEA* in *Arabidopsis*^19^. *E3* and *E4* are homologs of *phytochrome A* (*PHYA*) and encode the photoreceptors *GmPHYA3*^20^ and *GmPHYA2*^21^, respectively. The genome of soybean, an ancient tetraploid, contains genes with multiple copies. These copies may promote neo- or sub-functionalization, which facilitates the evolution of distinct functions in photoperiodic regulation^22^. Three *GmFT*s (i.e., *GmFT2a*, *GmFT5a*, and *GmFT4*) regulate flowering in soybean^23^,^24^,^25^. *GmFT2a* corresponds to previously identified flowering locus *E9*^17^. In addition, overexpression of several *CO* homologs, i.e., *GmCOL1a*, *GmCOL1b*, *GmCOL2a*, and *GmCOL2b*^26^,^27^, influences flowering. Overexpression of *GmCOL1a* could also down-regulate *E1* expression^27^. However, the precise role of soybean *CO* homologs in flowering must be verified through reverse genetic approaches or complementation test. Several *FT*-like genes have been identified in other legumes. Among these genes, *FTb2* in pea (*Pisum sativum*)^28^ and *MtFTa1* in Medicago (*Medicago truncatula*)^29^ regulate flowering. *MtFTa1* is also a key regulator of flowering time in Medicago. *FT* genes possibly possess a conserved function in photoperiodic flowering in legumes. *CO* homologs have also been identified in legumes, such as common bean (*Phaseolus vulgaris*)^30^, pea^31^, Medicago^32^,^33^, and *Lotus japonicus*^34^. Most *CO*-like genes in legumes are related to *Arabidopsis CO*-like genes than to *AtCO*, and these genes demonstrate an expression pattern distinct from that in *Arabidopsis*^35^. Although several *CO* homologs have been identified in Medicago, no solid evidence proves that *CO* homologs play a critical role in photoperiodic flowering in this species^32^,^33^. Therefore, the function of *CO*-like genes in regulating flowering in legumes remains unclear, whereas the main flowering time pathway in some leguminous species, e.g. pea, has been proven to be distinct from the *CO-FT* module in *Arabidopsis*.

Soybean is an important model plant used to elucidate photoperiodism in legumes^36^. *E1* is a major gene associated with flowering time and maturity and is located at the pericentromeric region; *E1* is intron-free and encodes a protein containing a putative bipartite nuclear localizing signal (NLS) and a domain distantly related to the plant-specific B3 domain (B3-like domain)^37^. Several members of the B3 superfamily directly or indirectly regulate flowering. *AtRAV1* in *Arabidopsis* also influences flowering time^38^. *TEMPRANILLO1* and *TEMPRANILLO2* genes can negatively regulate *FT* expression and floral transition in *Arabidopsis* through direct binding of the B3 domain to a consensus bipartite sequence element in the *FT* 5′-untranslated region^39^. In addition, several genes containing the B3 domain, such as *AUXIN RESPONSE FACTOR 1*^40^ and *LEAFY COTYLEDON2*, regulate plant growth and development^41^. The four major recessive alleles of *E1* identified in soybean are *e1-as* (a single missense point mutation), *e1-fs* (a 1 bp deletion leading to frame-shift), *e1-nl* (~130 kb deletion comprising the *E1* gene), and *e1-b3a* (three SNPs and 2 bp deletion in the middle of the B3 domain)^42^. *e1-as* is apparently a leaky allele and partially suppresses flowering in soybean^37^,^42^, contrary to the functional *e1-fs* and *e1-nl*. *E1* is expressed in a bimodal pattern, with higher expression in long-day (LD) conditions than in short-day (SD) conditions. *E1* is a putative transcription factor (TF) that negatively controls *GmFT2a* and *GmFT5a* to delay flowering under the background with functional *PHYA* genes (*E3E4*) and LD conditions. Zhai *et al.*^42^ proved that *E1* can positively regulate *GmFT4* to control flowering in soybean. Xu *et al.*^43^ revealed that, similar to *E1*, two *E1* orthologs, namely, *E1La* (*Glyma.04G156400.1*) and *E1Lb* (*Glyma.04G143300.1*), can control the onset of flowering. That *LjE1L* controls flowering was proposed based on the analysis of the polymorphism of *Lj5g3v2221340* (*LjE1L*, an ortholog of *E1*) among different wild *Lotus japonicus* species from various geographic locations in Japan^44^.

Fossil and molecular dating approaches indicated that legume originated ~60 million years ago (Mya)^45^,^46^ followed by speciation. The major legume species belong to two clades, namely, Hologalegina and Papilionoideae (Millettioid/Phaseoloid). Hologalegina, which includes Medicago, chickpea (*Cicer arietinum*), and *L. japonicus*, originated ~51 Mya, earlier than Millettoid/Phaseoloid (~45 Mya), which includes common bean, soybean, and pigeon pea (*Cajanus cajan*)^45^. Moreover, Medicago and soybean underwent two rounds of whole genome duplication (WGD) events; the older WGD occurred at ~58 Mya before *Glycine/Medicago* split^45^, and the recent WGD in soybean occurred at ~13 Mya^47^. The duplicated genes underwent sub- or neo-functionalization after the WGD, and some of these genes became pseudogenes^22^. Many highly syntenic regions were identified and characterized in Medicago, *L. japonicus*, common bean, and soybean^48^,^49^,^50^. The soybean genome exhibits higher conservation with the common bean genome than those with Medicago and *L. japonicus*. Therefore, whether *E1* homologs from legume species exert a function similar to or different from that of *E1* as a flowering repressor in soybean remains unknown.

In this study, we analyzed the protein structure and phylogenetic relationships among *E1* family genes identified in legumes. We also retrieved sequences of *E1* homologs in common bean and Medicago and performed functional analysis by overexpressing the homologs in soybean, *Arabidopsis*, and rice. Functional conservation and diversification among *E1* family genes of leguminous species were consistent with the divergence time of genes and lineage species in this plant group.

## Results

### Gene and protein structures of the *E1* family

**Figure 1:**
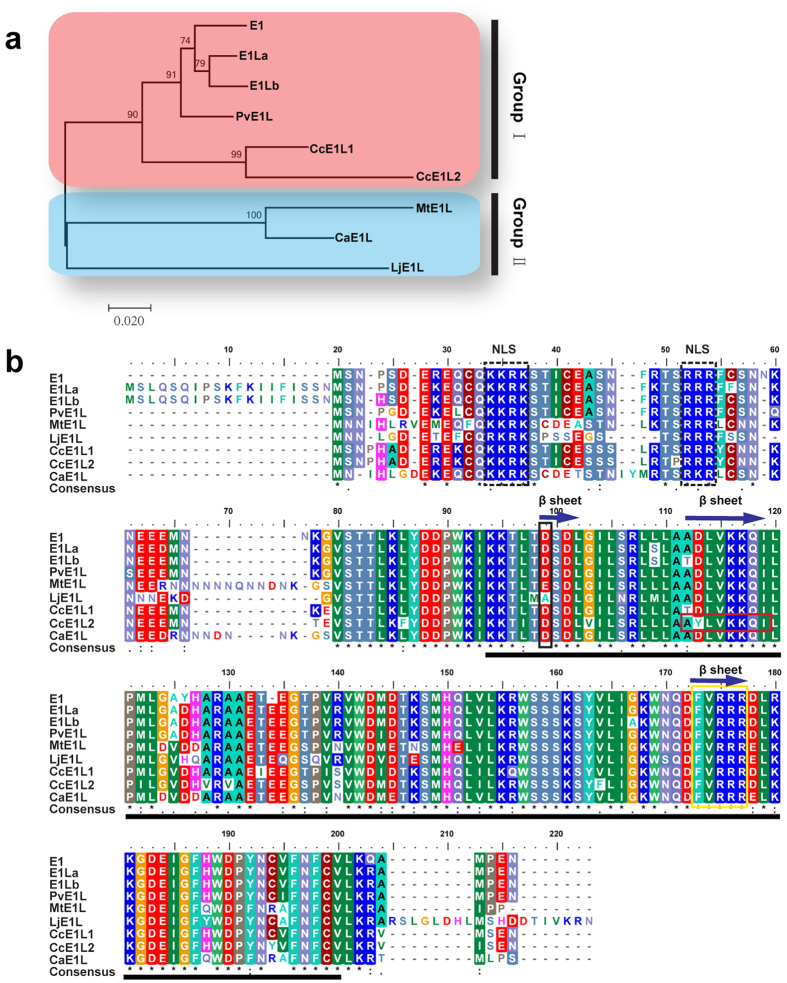
Phylogenetic relationship and sequence alignment of *E1* family genes. (**a)** Phylogenetic tree of *E1* family genes. The full length amino acid sequences of E1 family proteins were aligned using Clustal W and the phylogenetic tree was constructed using the Neighbor-joining method in MEGA 6.0 (Bootstrap = 1000). Two main groups, Group I and Group II, corresponding to genes from Millettioid/Phaseoloid and Hologalegina clade marked in red and blue. **(b)** Amino acid sequence alignment of nine *E1* family genes from legumes. The B3-like domain is underlined in black. Putative bipartite nuclear localization signals (NLS) are shown in dotted black boxes. The residues sequences shown in black and red boxes form putative extra helices in *MtE1L* and *LjE1L*, respectively. The residues in the yellow box correspond to the conserved helix of nine E1 family proteins. All putative helices are shown as blue arrows.

**Figure 2:**
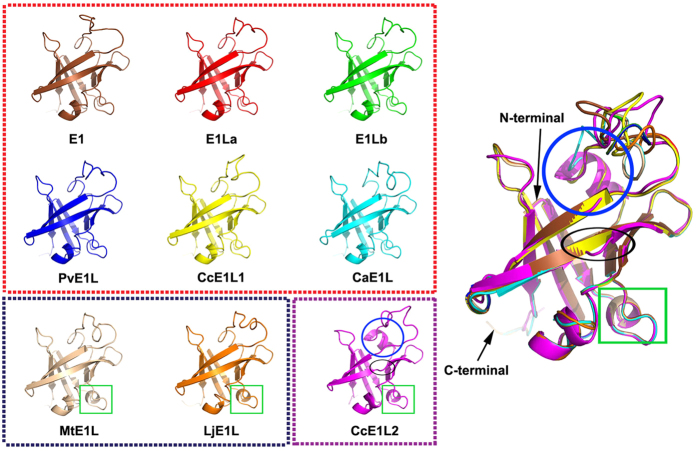
Putative 3D protein structures of *E1* family genes. Putative 3D protein structures of nine *E1* family genes from legumes. The structures of E1, E1La, E1Lb, PvE1L, CcE1L1 and CaE1L are displayed in the dotted red frame, whereas those of MtE1L and LjE1L are shown in the dotted blue frame with the extra helix shown in the green box. The structure of CcE1L2 is shown in the dotted purple frame with extra helices shown in the green box or blue circle. The separated position of one helix is shown in the black oval. The structures of the nine proteins are superimposed on the figure shown in the right-hand.

Eight gene products that are highly homologous [amino acid (aa) identity >60%] to *E1* genes were retrieved from Phytozome, NCBI, or Legume Information System (LIS). The eight genes, namely, *Phvul.009G204600* (*PvE1L*) from common bean, *C. cajan_45915* (*CcE1L1*) and *C. cajan_26468* (*CcE1L2*) from pigeon pea, *Ca_21849* (*CaE1L)* from chickpea, *Medtr2g058520* (*MtE1L*) from Medicago, *LjE1L* from *L. japonicas*, and *E1La* and *E1Lb* from soybean, along with E1, are referred to as *E1* family hereafter. Phylogenetic analysis of E1 family proteins in legume showed that all nine proteins divided into two main groups, namely Group I and Group II (Fig. 1a). The Group I consists of *E1*, *E1La*, *E1Lb*, *PvE1L*, *CcE1L1*, and *CcE1L2*, whereas the Group II consists of *LjE1L*, *MtE1L*, and *CaE1L*. We followed the annotation of Xia *et al.*^37^, that is, *E1* encodes a 174-aa protein, which was supported by the result of rapid amplification of cDNA ends (RACE). Although *Glyma.06G207800* was annotated at the *E1* locus in the version of Wm82.a2.v1 at Phytozome with 60 aa longer than *E1*, this finding has not been supported by experimental data. RACE analysis revealed that *E1La* and *E1Lb* encode 192-aa proteins^43^, which are 19 aa longer than that predicted by Xia *et al.*^37^. Both proteins shared high similarity (85%) to *E1* at the aa level. Six other *E1* homologs, namely, *PvE1L*, *CcE1L1*, *CcE1L2*, *CaE1L*, *MtE1L*, and *LjE1L*, putatively encoded proteins containing aa residues ranging from 173 (PvE1L) to 184 (MtE1L) and sharing 62% (MtE1L) to 91% (PvE1L) aa identity with E1. All *E1* family genes were predicted to contain no intron but a putative bipartite NLS at 34–55 aa residue in the consensus sequence; this bipartite NLS is composed of the KKRK and RRR basic domains at either ends separated by 14 aa residues. Moreover, all genes were predicted to contain a B3-like domain (Fig. 1b). The alignment showed that most residues were highly conserved (Fig. 1b). In addition, three-dimensional (3D) structures of all *E1* family proteins were predicted to possess a structure similar to that of the DNA-binding protein RAV1 (SWISS-MODEL template library ID: 1wid.1.A). We predicted that all nine E1 family proteins contain the seven basic beta sheets and loops, along with 1–3 helices. However, the third beta sheet in CcE1L2 was predicted to separate into two beta sheets (Fig. 2). Moreover, six protein structures (E1, E1La, E1Lb, PvE1L, CcE1L1, and CaE1L) of the *E1* family possessed only one helix of a sequence stretch “FVRRR”. By contrast, MtE1L or LjE1L contained an extra helix of “ESDL” or “ASDL” at the position of “DSDL” in most proteins. CcE1L2 also possessed extra two helices, namely, “DSDL” and “AYLVKKQI” (Figs 1b and 2).

### Expansion pattern and microsynteny of the *E1* family genes

*E1* family genes, except for *CcE1L1* and *CcE1L2*, were annotated on six chromosomes in different genomes (Supplementary Fig. S1). *E1La* and *E1Lb* possibly originated from segmental duplication because both genes were annotated on chromosome 4 and located at approximately 10 Mb apart (Supplementary Fig. S1). To examine whether *E1* family genes are involved in segmental duplication, we retrieved the syntenic blocks of individual *E1* homologs from the Plant Genome Duplication Database (PGDD). Nonsynonymous (*Ka*) and synonymous (*Ks*) substitution rates were then used to estimate date of divergence. The *Ka/Ks* ratio of the segmental pairs in soybean varied from 0.1736 to 0.2089 (ratio < 1) (Table 1), indicating that *E1*, *E1La*, and *E1Lb* underwent negative or purifying selection. The soybean genome underwent two WGD rounds, which occurred ~58 and ~13 Mya^47^. The divergence time between *E1* and *E1La*/*E1Lb* can be traced back to 10.6 and 8.8 Mya, respectively (Table 1), which is possibly associated with the recent WGD in soybean. The divergence time between *E1* and *PvE1L* was also estimated to have occurred 14.1 Mya. By contrast, the divergence time between *E1* and *MtE1L* could be traced back to 62.9 Mya (Table 1). Among the *E1* family members in non-soybean legumes, *PvE1L* displayed the closest genetic relationship with *E1*. In addition, estimated divergence times between *E1* and *CcE1L1*/*CcE1L2* are 28.3 and 28.1 Mya, respectively. Similarly, the divergence time between *E1* and *LjE1L*/*CaE1L* are 60.8 and 88.5 Mya, respectively (Table 1). The evolution rates may not be inaccurate because of differences among species or even among the distinct locations of genes in a chromosome. Nevertheless, these predicted results revealed the sequential evolutionary relationship of these genes. The *E1* family genes can be divided into two main groups, Group I and Group II, based on the estimated divergence times (Supplementary Table S1), corresponding to the Millettiod/Phaseoloid and Hologalegina branch, respectively^51^. More conserved syntenic blocks were also observed between the region of the *E1* and that of *E1La* or *E1Lb* than that between the region of *E1* and that of *PvE1L*, *MtE1L*, or *CaE1L* (Supplementary Fig. S2). These results are consistent with expected decline in conservation with taxonomic distance. We could not retrieve any *E1* homolog in *L. japonicus* and *C. cajan* from the CoGe server for syntenic analysis.

### Effects of *E1* and *PvE1L* overexpression on flowering time and plant growth in soybean

Xia *et al.*^37^ reported that the expression of an additional copy of *E1* driven by its native promoter in an early-flowering cultivar Kariyutaka (*E1*) can significantly delay flowering. In this study, we investigated the functional complementation of the *E1* gene under recessive *e1-as* allele genetic background. We simultaneously transformed the *PvE1L* gene into the soybean to test whether *PvE1L* can control the onset of flowering. All transformations were performed using the soybean cultivar Dongnong 50 (WT-DN) carrying the *e1*-*as* allele.

**Figure 3:**
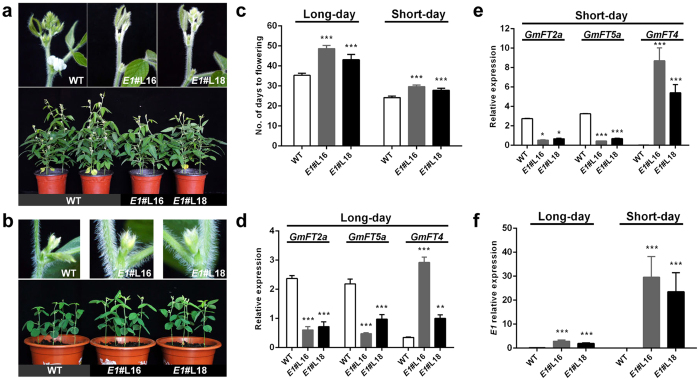
Overexpression of *E1* in soybean delayed flowering under LD and SD conditions. Flowering phenotype of wild-type Dongnong 50 (WT-DN) and two *E1* transgenic lines under LD (**a**) and SD (**b**) conditions. Annotation of cultivar or lines is indicated underneath. Close-up images that correspond to each pot show the axils of trifoliate leaves at approximately 35 and 24 DAE under LD and SD conditions, respectively. Two *E1* transgenic lines (*E1*#L16 and *E1*#L18) did not display flowers or flower buds under LD conditions. (**c)** Days to flowering in WT-DN plants and *E1* transgenic lines. Herbicide-resistant T_3_ plants of two transgenic lines were grown for flowering time investigation. Values represent the average of six replications +s.d. Similar results were observed in three separate experiments. Statistical significance was determined using Dunnett’s post hoc test after a significant one-way ANOVA. (**d**,**e)** Expression of *GmFTs* (*GmFT2a*, *GmFT5a*, and *GmFT4*) in the third fully expanded trifoliate leaves of WT-DN and *E1* transgenic lines under LD and SD conditions, respectively. (**f)** Expression of *E1* in *E1* transgenic lines. Relative expression levels were analyzed by quantitative RT-PCR and normalized to *TUA5*. Values represent the average of three biological replicates +s.d. *, **, and ***indicate significant differences between transgenic lines and WT-DN plants at P < 0.05, P < 0.01, and P < 0.001, respectively.

**Figure 4:**
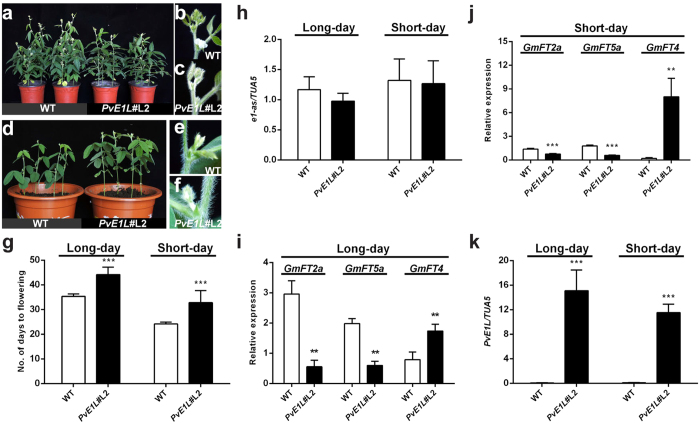
Ectopic overexpression of *PvE1L* in soybean delayed flowering under LD and SD conditions. Flowering phenotype between WT-DN lines and the *PvE1L* (*PvE1L*#L2) transgenic issue under LD (**a–c)** and SD (**d–f)** conditions, respectively. (**a**,**d)** WT-DN plants exhibiting flowers and pods at the axils of trifoliate leaves at approximately 35 and 24 DAE under LD and SD conditions, respectively. Close-up images of **(b)** WT-DN plant with flowers, and (**c)** one *PvE1L* transgenic plant with flower buds under LD contidions. Close-up images of (**e)** WT-DN plants with a pod (approximately 8 mm in length), and (**f)** *PvE1L*#L2 with flower buds at the axils of trifoliate leaves under SD conditions. (**g)** Days to flowering in WT-DN line and *PvE1L*#L2. Herbicide-resistant T_3_ plants of *PvE1L*#L2 were grown for flowering time investigation. Values represent the average of six replicates + s.d. P-value was determined using two-tailed Students’ *t*-test. Similar results were observed in three separate experiments. (**h)** Expression of native *e1-as* in WT-DN and *PvE1L* overexpression lines under LD and SD conditions. (**i**,**j)** Expression of *GmFTs* (*GmFT2a*, *GmFT5a*, and *GmFT4*) in the third fully expanded trifoliate leaves of WT-DN and *PvE1L* transgenic lines under LD and SD conditions, respectively. (**k)** Expression of *PvE1L* in WT-DN and transgenic plants. Relative expression levels were analyzed by quantitative RT-PCR and normalized to *TUA5*. Values represent the average of three biological replicates +s.d. *, **, and ***indicate significant differences between transgenic lines and WT-DN plants at P < 0.05, P < 0.01, and P < 0.001, respectively.

Two independent *E1* complementation lines (*E1*#L16 and *E1*#L18) and one *PvE1L* overexpression line (*PvE1L*#L2) were obtained, We painted the leaves with 160 mg L^−1^ glufosinate to test herbicide resistance, and the resistant individuals were segregated from the non-resistant individuals in an approximately 3:1 ratio (Supplementary Table S2). Herbicide-resistant T_1_ and T_2_ plants of transgenic *E1* and *PvE1L* lines exhibited late-flowering phenotype, Statistical analysis of flowering time data of herbicide-resistant T_3_ plants of transgenic lines revealed that two *E1* transgenic lines and *PvE1L*#L2 flowered significantly later than the WT-DN plants in both LD (Figs 3a and 4a–c) and SD (Figs 3b and 4d–f) conditions. The WT-DN plants flowered at 35.3 days after emergence (DAE) under LD conditions and 24.2 DAE under SD conditions. In addition, *E1*#L16 and *E1*#L18, which are two *E1* transgenic lines, flowered at 48.7 and 43.2 DAE under LD conditions and at 29.7 and 27.8 DAE under SD conditions. These results indicate that *E1* overexpression delays the onset of flowering. The *PvE1L* transgenic line also flowered at a significantly later time (44.2 DAE and 33.2 DAE under LD and SD conditions, respectively) compared with the WT-DN plants (35.3 and 24.2 DAE under LD and SD conditions, respectively) (Figs 3c and 4g). *PvE1L* overexpression had no effect on the expression of native *e1-as* in *PvE1L*#L2 (Fig. 4h). Moreover, the expression levels of *GmFT2a* and *GmFT5a* were significantly reduced, whereas *GmFT4* expression was significantly induced (Figs 3d,e and 4i,j) in transgenic lines when *E1* (Fig. 3f) and *PvE1L* (Fig. 4k) were overexpressed under LD and SD conditions. These results are consistent with previous findings^25^,^37^. *E1* complementation test results further proved that *E1* is a flowering repressor in soybean. *PvE1L* also demonstrated a conserved physiological function as a flowering repressor. Interestingly, the *PvE1L* gene was annotated in a physical position, where two most significant QTLs (*df9.1* and *df9.*2) for flowering time were mapped^52^ (Supplementary Fig. S1); Thus, *PvE1L* possibly suppresses flowering by regulating the expression of *FT*-like genes in common bean. In this study, we propose that *PvE1L* plays a key role in modulating flowering time in common bean, although this assumption requires more actual evidence confirmation.

In addition, overexpression of *E1* and *PvE1L* in soybean also resulted in several morphological changes, e.g., plant height and number of nodes (Supplemental Figs S3a–d and S4a–d). WT-DN plants in LDs were 66.3–73.1 cm tall with 18.3–19.0 nodes, whereas the *E1*#L16, *E1*#L18, and *PvE1L*#L2 were significantly taller (27%, 24% and 15% increase, respectively) than WT-DN plants. This characteristic was due to the increase in the number of nodes (3.7, 3.3 and 2.4 nodes more, respectively) because internode length was not significantly different from WT-DN plant (Supplemental Figs S3c–e and S4c–e). Although all lines appeared shorter than under LD conditions, similar trends in plant height and node number between transgenic *E1*/*PvE1L* lines and WT-DN were observed under SD conditions (Supplemental Figs S3 and S4).

### Functional characterization of *MtE1L* in Medicago and soybean

**Figure 5:**
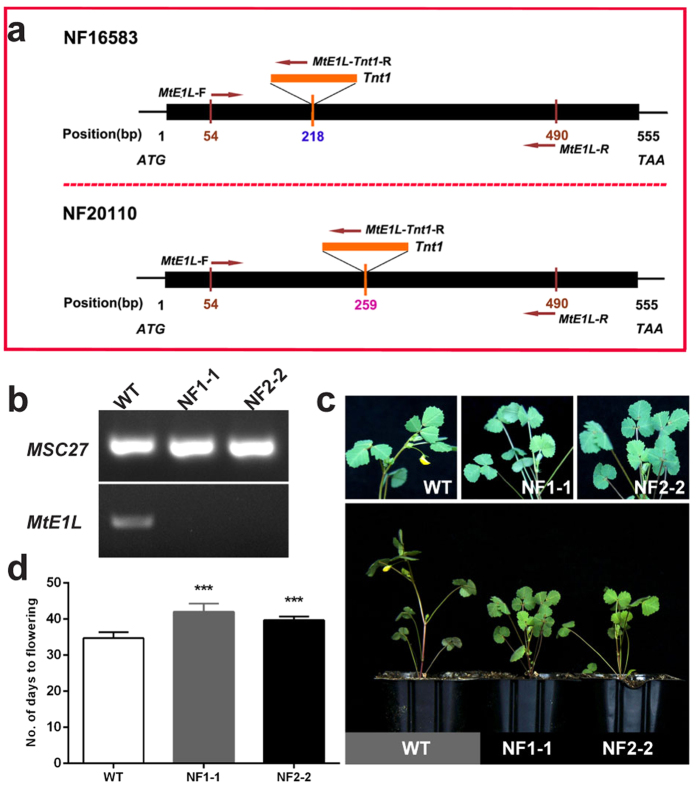
Medicago *mte1l* mutants were late-flowering. **(a)** Two independent *Tnt1* mutations occurring at different positions in *MtE1L*. (**b)** Semi-quantitative RT-PCR analysis of *MtE1L* expression in wild-type cultivar R108 (WT-Mt) and two mutants lines under LD conditions. (**c)** Flowering phenotype of WT-Mt and two *mte1l* lines, NF1-1 and NF2-2, in LDs. Seedlings were vernalized for 10 days and then grown under LD conditions. Close-up of WT-Mt plant showing flowers at approximately 35 DAE. Two homozygous mutant lines did not exhibit flowers. (**d)** Number of days to flowering in WT-Mt and two homozygous mutant lines. Values represent the average of twelve replicates +s.d. Similar results were observed in two separate experiments. Statistical significance was determined using Dunnett’s post hoc test after a significant one-way ANOVA. ***indicates significant difference between mutant lines and WT-Mt plants at P < 0.001.

Two individual mutant lines, namely, NF16583 (NF1) and NF20110 (NF2), containing *Tnt1* retrotransponson-tagged insertions occurring at different positions in *MtE1L*, were obtained from the *M. truncatula* Mutant Database (http://medicago-mutant.noble.org/mutant/). Four homozygous mutant plants, namely, NF1-1 and NF1-2 from NF1 line and NF2-2 and NF2-4 from NF2 line, were verified by PCR analysis (Supplementary Fig. S5). Sequencing confirmed the insertion positions to be at 218 and 259 bp in the open reading frame of NF1 and NF2, respectively (Fig. 5a). In this study, seedlings from the homozygous mutant lines, NF1-1 and NF2-2, were used to investigate flowering time. No *MtE1L* expression was detected in both homozygous mutants (Fig. 5b), suggesting that the expression or function of *MtE1L* in these mutant lines was interrupted by *Tnt1* insertion. Moreover, the two homozygous mutant lines flowered at 42.0 (NF1-1) and 39.7 (NF2-2) DAE, later than that of WT-Mt (34.7 DAE) (Fig. 5c,d). Similar phenotype of flowering time between the two mutant lines and WT-Mt was observed in the second independent experiment. Thus, both mutant lines demonstrated late flowering phenotype. Two F_2_ populations of NF1-2-4 (♂) × R108 (♀) and NF2-4-4 (♂) × R108 (♀) were constructed to further confirm whether the mutation was the causal factor that led to the variation in flowering time. Fifteen plants of NF1-2-4 and thirteen plants from NF2-4-4 derived F_2_ plants were genotyped and phenotyped in the first trial. The corresponding segregation ratios of wild-type: heterozygous: homozygous are 4:7:4 and 3:6:4, both of which were approximately consistent with the 1:2:1 of Mendelian inheritance. The flowering time of homozygous mutants was significantly later than those of WT-Mt or heterozygous mutants in both F_2_ populations (P < 0.05, univariate ANOVA)(Supplementary Fig. S6). This phenomenon indicated that both mutations in *MtE1L* were significantly associated with flowering time.

**Figure 6:**
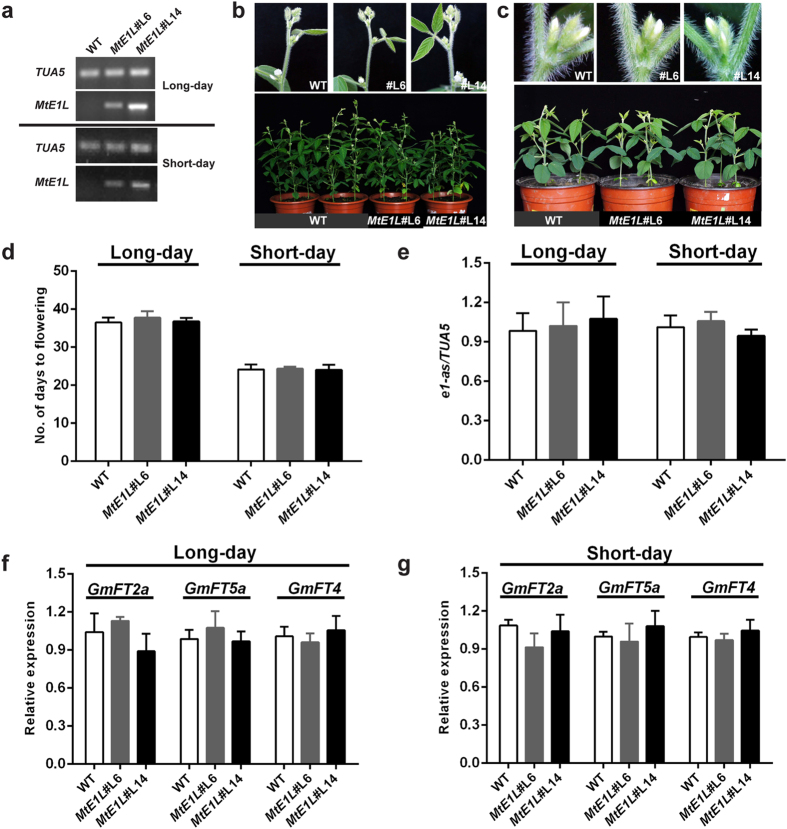
Ectopic overexpression of *MtE1L* in soybean exerted no noticeable effect on flowering time under LD and SD conditions. **(a)** Semi-quantitative RT-PCR analysis of *MtE1L* in two transgenic lines under LD and SD conditions. (**b**,**c)** Flowering phenotype of WT-DN and two *MtE1L* transgenic lines (*MtE1L*#L6 and *MtE1L*#14). All lines flowered at ~37 and 24 DAE under LD and SD conditions, respectively. (**d)** Days to flowering in WT-DN plants and *MtE1L* transgenic lines. Herbicide-resistant T_3_ plants of two transgenic lines were grown for flowering time investigation. Values represent the average of approximately six replicates +s.d. Similar results were observed in three separate experiments. Statistical significance was determined using Dunnett’s post hoc test after a significant one-way ANOVA. Expression of **(e)** native *e1-as* and **(f,g)** *GmFTs* (*GmFT2a*, *GmFT5a*, and *GmFT4*) in the third fully-expanded trifoliate leaves of *MtE1L* transgenic lines and WT-DN plants under LD and SD conditions. Relative expression levels were analyzed by quantitative RT-PCR and normalized to *TUA5*. Values represent the average of three biological replicates +s.d.

These results suggested that *MtE1L* may regulate flowering in Medicago. To investigate whether ectopic overexpression of *MtE1L* in soybean affects flowering time, we transformed *MtE1L* into Dongnong 50. Two soybean transgenic lines (*MtE1L*#L6 and *MtE1L*#L14) showed high *MtE1L* expression, whereas no *MtE1L* expression was detected in WT-DN lines (Fig. 6a). Meanwhile, progenies of both transgenic lines were segregated when tested for herbicide resistance. The resistant to non-resistant individuals were consistent with 3:1 of Mendelian inheritance (Supplementary Table S2). Two transgenic *MtE1L* soybean lines flowered at 36.3–37.2 and 24.0–24.3 DAE under LD and SD conditions not significantly different from that of the WT-DN plants (36.5 and 24.2 DAE under LD and SD conditions) (Fig. 6b–d). In addition, the expression levels of native *e1*-as, *GmFT2a*, *GmFT5a*, and *GmFT4* in transgenic plants were consistent with those in WT-DN plants both under LD and SD conditions (Fig. 6e–g). The significant difference of WT-DN plants in flowering time between LD or SD conditions revealed that the soybean cultivar Dongnong 50 was sensitive to photoperiod. Previous study showed that *e1-as* represents a partial functional allele that controls flowering in soybean^42^. Thus, the ectopic overexpression of *MtE1L* in soybean did not affect flowering in soybean. Taken mutant analysis and transgene in soybean together, we propose the *MtE1L* gene is a flowering promoter in Medicago, contrary to the *E1* or *PvE1L* genes as flowering repressor.

The *MtE1L* transgenic lines exhibited a dwarf phenotype with fewer nodes and shorter internode length compared with that of WT-DN plants under LD or SD conditions (Supplemental Fig. S7a,b). Transgenic *MtE1L* lines in LD conditions were significantly shorter (25% to 32% decrease) than WT-DN plants (73.4 cm tall). This characteristic was contributed by a combination of 2.7–3.3 fewer nodes and 0.6–0.7cm shorter in internode length than WT-DN plants possessing 19.0 nodes with 4.0 cm long of internode length (Supplemental Figs S7c–3e). All lines under SD conditions were shorter than that under LD conditions, but similar variations in plant height, node number, and internode length between *MtE1L* transgenic lines and WT-DN were observed (Supplemental Fig. S7).

### Effect of ectopic overexpression of *E1* on flowering in *Arabidopsis* and rice

Three *Arabidopsis E1* overexpression lines (*E1*#L3, *E1*#L10, and *E1*#L11) and three vector-only transgenic lines (VC#L2, VC#L4, and VC#L7) were obtained (Supplementary Fig. S8a,b). All three *E1* overexpression lines demonstrated high levels of *E1* expression (Supplementary Fig. S8b). In addition, *E1* overexpression lines flowered at 28.4–29.7 DAE, which was not significantly different from those of WT-At or VC lines under LD conditions (28.7–29.5 DAE) (Supplementary Fig. S8c). *E1* overexpression lines, namely, WT-At and VC lines, also displayed similar number of rosette leaves (13.5–14.9 on average) when flowering (Supplementary Fig. S8d). The ectopic overexpression of *E1* during flowering time apparently did not cause phenotypic changes in *Arabidopsis*. Similar results were observed in a separate experiment. Expression profiles of *AtFT* genes and *AtCO* were not significantly different between WT-At and all transgenic lines (Supplementary Fig. S8e).

Two rice *E1* overexpression transgenic lines, namely, *E1*#L2 and *E1*#L4, were obtained (Supplementary Fig. S9a). All lines, including wild-type rice (WT-Os) and two *E1* transgenic lines, required 52.1–52.8 days to flower under SD conditions (Supplementary Fig. S9b,c). Similar phenotypes of flowering time of all lines were observed in an independent experiment. Similar to that observed in *Arabidopsis*, the expression profiles of *OsHd1* and *OsHd3a*, which are respective *AtCO* and *AtFT* orthologs in rice, were not significantly different between WT-Os and transgenic rice lines (Supplementary Fig. S9d).

## Discussion

The legume family consists of crops that serve as primary sources of plant proteins for food and feed. Flowering is one of the major agronomic traits of legumes and has been the focus of fundamental and applied studies. Flowering regulation pathway in soybean is distinct from that in model plants, such as *Arabidopsis* and rice. Moreover, *E1* is a key gene in the gene regulatory network of flowering in soybean. The product of this gene is a negative regulator of the photoperiod response pathway. However, the exact molecular function and evolutionary history of the *E1* gene remains to be elucidated.

Most TF proteins consist of two domains, namely, a DNA-binding domain that binds to the promoter of target genes and a transcription activation (or suppression) domain that activates or suppresses transcription of target genes. *E1* has been proven that *E1* as a negative regulator of the flowering pathway^37^. However, the mechanism of action of the *E1* family genes remains unclear. TFs are often associated with conserved functions across distantly related species. However, E1 family proteins appear to be only effective in closely related species. Given that *E1* family genes are only recovered from legume species, the failure to detect any phenotype caused by overexpression of *E1* in *Arabidopsis* and rice suggests that the exogenous *E1* gene is independent of the regulation networks of photoperiodic flowering in *Arabidopsis* and rice. *E1* function within legume might be, conserved between soybean and common bean, only diverging for ~14 Mya, which is a rather short distance based on evolutionary perspective. In this study, ectopic overexpression of *PvE1L* gene in soybean resulted in similar flowering and morphologic changes to transgenic *E1* lines. The extent by which the *PvE1L* gene contributes in the control of flowering time in common bean needs further investigation. No flowering phenotype was observed, when *MtE1L* was overexpressed in soybean. This result suggests that the *E1* network may differ between the two species that diverged for ~60 Mya. Alternatively, more soybean varieties should be tested to verify the result from this particular cultivar. Nevertheless, *E1* could evidently perform distinct functions in the same family of plants. Therefore, *E1* represents a TF that evolved rapidly.

In this study, we recovered a total of nine *E1* family genes from six legume species. *E1* is a single copy gene in four species and duplicated in soybean and pigeon pea, suggesting that *E1* duplication is not a frequent event. Sequence divergence among the orthologs of *E1* indicates that the initial *E1* gene can be tracked back prior to the legume speciation. The two *E1* family genes in pigeon pea have separated for ~3.9 Mya (Supplementary Table S1). Three *E1* copies, namely, *E1*, *E1La*, and *E1Lb* were found in soybean. These copies were derived from two duplication events. The first duplication event engendered *E1* and the ancestor of *E1La* and *E1Lb*, which occurred between 8.8 Mya to 10.6 Mya. The second duplication event led to the generation of *E1La* and *E1Lb*, which occurred at ~3 Mya because of segmental duplication in chromosome 4. If neither *E1* duplication events were due to genome wide duplication, the amplification of *E1* gene has been suggested to present a selective advantage in soybean. The differentiation in expression patterns, in conjunction with the combination of functional, partially functional, and non-functional copies of the *E1* gene, may have enabled soybean to adapt in various geological locations.

These genes apparently perform different functions in flowering, although *E1* family genes in legume seem to have the same origin. *E1, E1La and E1Lb*^43^ in soybean suppress flowering, whereas *MtE1L* in Medicago appears to promote flowering. The distinct function was probably due to the presence of an extra helix in the proteins (Fig. 2). Interestingly, the extra helix was present in MtE1L from *M. truncatula*, but not in E1. The association between E1 structure and flowering physiology might be an interesting topic for further studies. From a parsimony perspective, the evolution of different forms of TFs from the same ancestral protein is efficient for “turning on” or “turning off” the same set of target genes to fulfill the photoperiod requirement in different plants or in different seasons of the same plant. The two *E1* copies in pigeon pea that only diverged for ~3.9 Mya support this notion. Interestingly, the predicted product from *CcE1L2* contained the extra helix, whereas that from *CcE1L1* was devoid of the extra helix. Moreover, *K*_a_/*K*_s_ value between the gene pair was almost 1 (0.94, Supplementary Table S1), implying the lack of purifying selection between two genes or evolution of different functions. We infer that both copies may exert different functions considering the distinct structures between the two concurrent copies of *E1* homologs in pigeon pea. Nevertheless, this result provides a good example to investigate the functional diversity in relation to structural variation.

**Figure 7:**
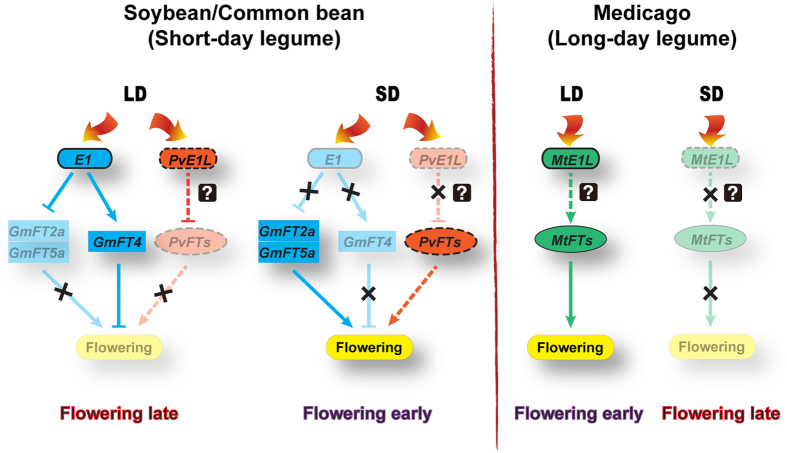
Proposed flowering regulation networks in legumes. Genes from soybean, common bean and Medicago are marked blue, red and green, respectively. Arrows represent stimulation of gene expression; T-shaped symbols represent inhibition of gene expression; Potential interactions are connected in dotted line. Symbol × represent the negation of promotion/inhibition. The deduced interactions were indicated by question marks.

The transition from vegetative to reproductive growth is a complex process that requires coordination among numerous genes, as well as the redistribution of resources between reproductive and vegetative organs. Consequently, late flowering is logically associated with increased plant height (see Supplementary Figs S3 and S4). Whether the reduced plant size accompanying early flowering is a result of reduced available resources for growth or an intentional inhibition of growth occurs to ensure flower development is a key question in the relationship of flowering development and growth. No flower phenotype was observed when *MtE1L* was overexpressed in soybean. This finding is unexpected because the relevant gene seemed to function in Medicago. More surprisingly, the dwarf phenotype was observed in the overexpression lines. This observation clearly indicates that the retarded growth was not a consequence of early flowering. Instead, this type of growth might be a part of the *MtE1L* regulation. The role and mechanism in flowering and plant development of *MtE1L*, and other members of the *E1* family merit further studies. Taken together, putative molecular networks of photoperiodic flowering regulation are proposed in legumes according to variations in photoperiodic response (Fig. 7). Networks of photoperiodic flowering regulation are conserved in short-day plants soybean and common bean, in which *E1* or *PvE1L* is highly expressed and suppresses the expression of the florigen genes *GmFT2a/5a* or *PvFTs* under LD conditions. However, under SD conditions, the expression of both genes is suppressed, and the florigens are upregulated. On the other hand, in long-day plant Medicago, the function of *MtE1L* is shifted to flowering promoting. However, in the proposed flowering networks in legume, some putative interactions need to be tested or confirmed in the future study, e.g. how *PvE1L* and *MtE1L* genes regulate florigen genes in their native species. Nevertheless, the functional diversification of *E1* family genes revealed in this study and further characterization of flowering networks will provide insights in natural selection, domestication of important traits as well as genome evolution in legume.

## Methods

### Sequence collection, protein structure, and phylogenetic analysis

The E1 protein sequence was used to search for *E1* homologs at NCBI (http://www.ncbi.nlm.nih.gov/), Phytozome v10.3 (http://phytozome.jgi.doe.gov/pz/portal.html), and Legume Information System (LIS, http://www.legumeinfo.org/). We retrieved general information, such as position in genome, coding sequence (CDS), and protein sequence, of nine highly similar homologs. In silico 3D protein structures of nine E1 family proteins from *G. max*, *P. vulgaris*, *C. cajan*, *C. arietinum*, *M. truncatula*, and *L. japonicas* were generated using SWISS-MODEL^53^ online modeling server and visualized with PyMOL.

The online NCBI conserved domain database (http://www.ncbi.nlm.nih.gov/cdd) was used to extract the protein sequences of the B3-like domain for phylogenetic analysis. Multiple sequence alignments were performed using the Clustal W program with default parameters in MEGA v6.0 with some manual editing. An unrooted neighbor-joining phylogenetic tree was constructed with a 1000 bootstrap repetition. Multiple sequences alignment of nine full-length E1 family protein sequences was generated using Clustal X v1.81 software with default parameters.

### Microsynteny between chromosome segments encompassing *E1* or its homologs from legumes

The syntenic blocks of each gene were searched against the PGDD to determine whether *E1* or its homologs are located in the regions involved in segmental duplication. *K*_a_/*K*_s_ and date of duplication occurrence were estimated using a previously reported method^54^. Pairwise alignments of CDS without the stop codon of duplicated genes were performed using the MUSCLE program in MEGA6.0 with default parameters. Phylogenetic tree was constructed using maximum likelihood method with *K*_a_/*K*_s_ value that was calculated online by CODEML program built in Phylemon 2 (http://phylemon2.bioinfo.cipf.es/evolutionary.html). The estimation of the date of the segmental duplication event was performed using the formula, *T*  =  *K*_s_/2*λ*, in which the mean synonymous substitution rate (*λ*) was set to 6.1 × 10^−9^ based on previous report for Fabaceae^55^.

The SynFind and GEvo tools at CoGe online server (https://genomevolution.org/CoGe/index.pl) were used for microsyntenic analysis. The segment (PAC.26299773), which represents a region that contained the *E1* sequence, was blasted using BlastZ built in SynFind against the genomes of legumes, namely, soybean, common bean, pigeon pea, chickpea, Medicago, and *L. japonicus*. Each segment containing *E1* family gene along with 200 kb flanking sequences at both sides without masking was used to identify genome evolution patterns. These segments were also used to construct the syntenic relationship map at GEvo with default parameters.

### Plant materials and growth conditions

Soybean cultivar Dongnong 50 was used to genetically transform *E1* and its homologs. Soybean plants were grown in a phytotron under constant temperature of 25 °C and average light fluence rate of 200–300 μmol m^−2^s^−1^ under either LD (16 h light/8 h dark) or SD (12 h light/12 h dark) conditions. Furongdidou, a common bean cultivar, was grown under LD conditions and was used to clone the *E1* homolog in common bean.

*Medicago truncatula* cultivar R108 and *Tnt1* insertion lines were obtained from the Medicago mutant database (http://medicago-mutant.noble.org/mutant/). Seeds were scarified, germinated overnight at room temperature (25 °C) on wet filter papers, and stored in Petri dishes at 4 °C in the dark for 10 days to allow seeds to germinate. Young seedlings were transplanted into the soil and grown in a phytotron under a constant temperature of 22 °C and average light fluence rate of 150 μmol m^−2^s^−1^ under LD (16 h light/8 h dark) conditions.

*A. thaliana* ecotype *Columbia* (*Col-0*) was used for genetic transformation and phenotypic analysis. Seeds were surface-sterilized with 70% ethanol and 10% sodium hypochlorite. Then, the seeds were rinsed several times with sterile distilled water and sown onto 1/2 MS medium plates for germination. Seedlings with 2–4 leaves were transferred into the soil and grown in a phytotron under constant temperature of 22 °C and average light fluence rate of 150 μmol m^−2^s^−1^ under LD (16 h light/8 h dark) conditions.

*Oryza sativa* L. ssp. *Japonica cv.* Longjing 11 was used for genetic transformation and phenotypic analysis. Seeds were harvested, dried at 37 °C, and then germinated in sterilized water under SD (10 h light/14 h dark) conditions. The plants were transplanted into soil under constant temperature of 26 °C and average light fluence rate of 200–300 μmol m^−2^s^−1^ under SD conditions when the shoots reached a height of approximately 5–6 cm.

### Genetic transformation in soybean, *Arabidopsis*, and rice

A series of plant overexpression and expression vectors were constructed for genetic transformation. All vectors contained the *bar* gene for selection. According to the nature of the intron-free *E1* family genes, *E1*, *PvE1L*, and *MtE1L* were amplified from Harosoy (soybean), Furongdidou (common bean) and R108 (Medicago), respectively, using gene-specific primers to which *Xba* I and *Sac* I cloning sites were manually introduced. The fragments of PCR products and *pTF101.1-GmFT2a* vector^23^ containing a cauliflower mosaic virus *35S* promoter (*CaMV 35S*) digested by *Xba* I*/Sac* I were assembled together to construct *pTF101.1*-*E1*, *pTF101.1*-*PvE1L*, and *pTF101.1*-*MtE1L*. These vectors were transferred into *Agrobacterium tumefaciens EHA101*. Meanwhile, another plant expression vector *pMDC123-E1*^37^, in which *E1* was driven by the *E1* native promoter, was transferred into *A. tumefaciens EHA105* and subsequently used for rice genetic transformation. *Agrobacterium*-mediated cotyledon-node explant method was used for soybean genetic transformation^56^, whereas floral dip method was used for *Arabidopsis* genetic transformation^57^, Moreover, *Agrobacterium*-mediated co-cultivation method was used for rice genetic transformation.

### Screening of transgenic plants

Transgenic soybean lines were screened by screening the T_0_, T_1_, T_2_, and T_3_ transgenic seedlings by daubing 160 mg L^−1^ glufosinate on unfolded preliminary leaves of seedlings. In addition, *E1/PvE1L/MtE1L*-specific primers (see Supplementary Table S3) were used for semi-quantitative RT-PCR and real-time RT-qPCR. Herbicide-resistant T_3_ seedlings were used for phenotypic and molecular analysis.

Seeds from transgenic *Arabidopsis* lines were surface-sterilized with 70% ethanol and 10% sodium hypochlorite. Then, the seeds were rinsed for 5–6 times with sterile distilled water, and sown onto 1/2 MS plates containing 25 μg L^−1 ^glufosinate for testing and germination. T_2_ lines with more than 95% herbicide-resistant seedlings (approximately 100 seedlings tested for each T_2_ line) were considered to be homozygous lines. T_3_ homozygous were confirmed by semi-quantitative RT-PCR with *E1*-specific primers and used for phenotype and expression analyses.

Seeds from transgenic rice lines were harvested and dried at 37 °C. Then, the seeds were germinated in water under SD (10 h light/14 h dark) conditions. The plants were transplanted into the soil under SD conditions when seedling shoots were about 5–6 cm height. T_0_, T_1_, and T_2_ transgenic lines were verified by semi-quantity RT-PCR with *E1*-specific primers (see Supplementary Table S3).

### Characterization and verification of Medicago mutants

A pair of gene-specific primers (*MtE1L*-F/*MtE1L*-R) and a pair of *MtE1L-Tnt1* combined primers (*MtE1L*-F/*MtE1L-Tnt1*-R) were used to identify *Tnt1* insertions using PCR^58^. PCR products were purified and sequenced. The primers used in genetic screening can be found in Supplementary Table S3. We constructed two populations of NF1-2-4 (♂) × R108 (♀) and NF2-4-4 (♂) × R108 (♀) to further confirm that the mutation was the causal factor determining flowering time. The F_2_ populations were developed from seeds of self-pollinated F_1_ plants. Fifteen individuals of NF1-2-4 and thirteen individuals of NF2-4-4 derived F_2_ populations were used for genotyping and phenotyping. Data were statistically analyzed (univariate ANOVA) for correlation using the GraphPad Prism 6 software (GraphPad Software Inc., San Diego, CA).

### Phenotypic observation of plants overexpressing *E1, PvE1L* and *MtE1L*

Six plants each from WT-DN and T_3_ herbicide resistant soybean plants were used to investigate flowering time. Six plants from each line were used for growth habit investigation under both LD and SD conditions. The flowering time observed or investigations were performed three times (approximately six plants per instance). Flowering time was recorded for days from emergence to the first open flower at any node on the main stem (R1). WT-At plants and T_3_ homozygous lines were used to investigate flowering time. Flowering time and total number of rosette leaves were recorded for each line when flowers emerged on the main stem. Approximately 40 plants for each line were recorded and two independent experiments were carried out. WT-Os and T_2_ overexpression lines in rice were used to investigate heading time. Heading date was recorded when a panicle was just coming out. Eight rice plants for each line were assessed and two independent experiments were carried out. Two-tailed Student’s *t*-test was used to compare single transgenic line with WT, whereas Dunnett’s post hoc test was used after a significant one-way ANOVA between multiple transgenic lines with WT using GraphPad Prism 6 software (GraphPad Software Inc., San Diego, CA).

### RNA isolation and quantitative real-time PCR (RT-qPCR) analysis

Total RNA of leaves were extracted as mentioned previously. Reverse transcription was performed with 2 μg of RNA through *TransScript*^®^ One-Step gDNA Removal and cDNA Synthesis SuperMix (TransGen, Beijing, China) according to the manufacturer’s instruction. RT-qPCR was performed on the LightCycle 96 system (Roche, Switzerland) using SYBR Green Master Mixture (TransGen, Beijing, China) according to the manufacturer’s instruction. Soybean *TUA5*, *Arabidopsis AtActin2*, or rice *OsUbi5* was used as an internal control for normalization. PCR cycling conditions were set up as in the following program: 94 °C for 30 s, followed by 40 cycles of 94 °C for 5 s and 60 °C for 30 s with fluorescence signal collection. Melting and cooling steps were set as default parameters. Relative gene expression levels were calculated using the 2^−ΔΔCt^ method. Three independent biological replicates were obtained and subjected to real-time PCR in triplicate. Raw data were standardized as described previously^59^. All primers for expression analysis are listed in Supplementary Table S3.

## Additional Information

**How to cite this article**: Zhang, X. *et al.* Functional conservation and diversification of the soybean maturity gene *E1* and its homologs in legumes. *Sci. Rep.* **6**, 29548; doi: 10.1038/srep29548 (2016).

## Supplementary Material

Supplementary Information

## Figures and Tables

**Table 1:** Substitution rate and date of divergence between *E1* and its homologs in legumes.

Gene pair	Ka	Ks	Ka/Ks	Estimated time (Mya)
*E1/E1La*	0.0225	0.1077	0.2089	8.8
*E1/E1Lb*	0.0225	0.1294	0.1736	10.6
*E1/PvE1L*	0.0302	0.1723	0.1752	14.1
*E1/MtE1L*	0.1473	0.7668	0.1921	62.9
*E1/LjE1L*	0.1114	0.7418	0.1501	60.8
*E1/CcE1L1*	0.0534	0.3454	0.1545	28.3
*E1/CcE1L2*	0.0748	0.3434	0.2179	28.1
*E1/CaE1L*	0.1359	1.0795	0.1259	88.5

Ka: nonsynonymous substitution rate; Ks: synonymous substitution rate; Mya: million years ago.
