# Supplementary material for: Functional conservation and diversification of the soybean maturity gene E1 and its homologs in legumes
Source: Sci Rep. 2016 Jul 13;6:29548. doi: 10.1038/srep29548 (PMC4942777; doi:10.1038/srep29548)
Supplement: Supplementary Information [file srep29548-s1.pdf]

Functional conservation and diversification of the soybean maturity gene *E1* and its homologs in legumes

Xingzheng Zhang[1,2#], Hong Zhai[1#], Yaying Wang[1,2], Xiaojie Tian[1,2], Yupeng Zhang[1,2], Hongyan Wu[1], Shixiang Lü[1,3], Guang Yang[1], Yuqiu Li[1,2], Lu Wang[1,2], Bo Hu[1], Qingyun Bu[1], Zhengjun Xia[1,*]

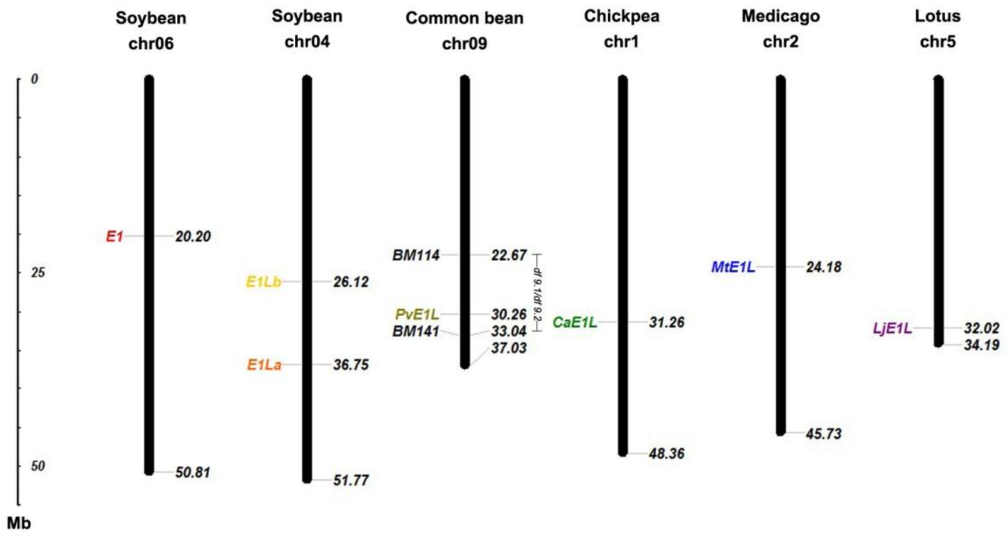

Supplementary Figure S1 Physical positions of *E1* family genes from legumes.

Physical positions of *E1* homologs in the genomes, except for *CcE1L1* and *CcE1L2* whose physical positions were unclear. The flanking markers (*BM114* and *BM141*) of *df9.1*/*df9.2* locus and their positions were shown on chr09 of common bean.

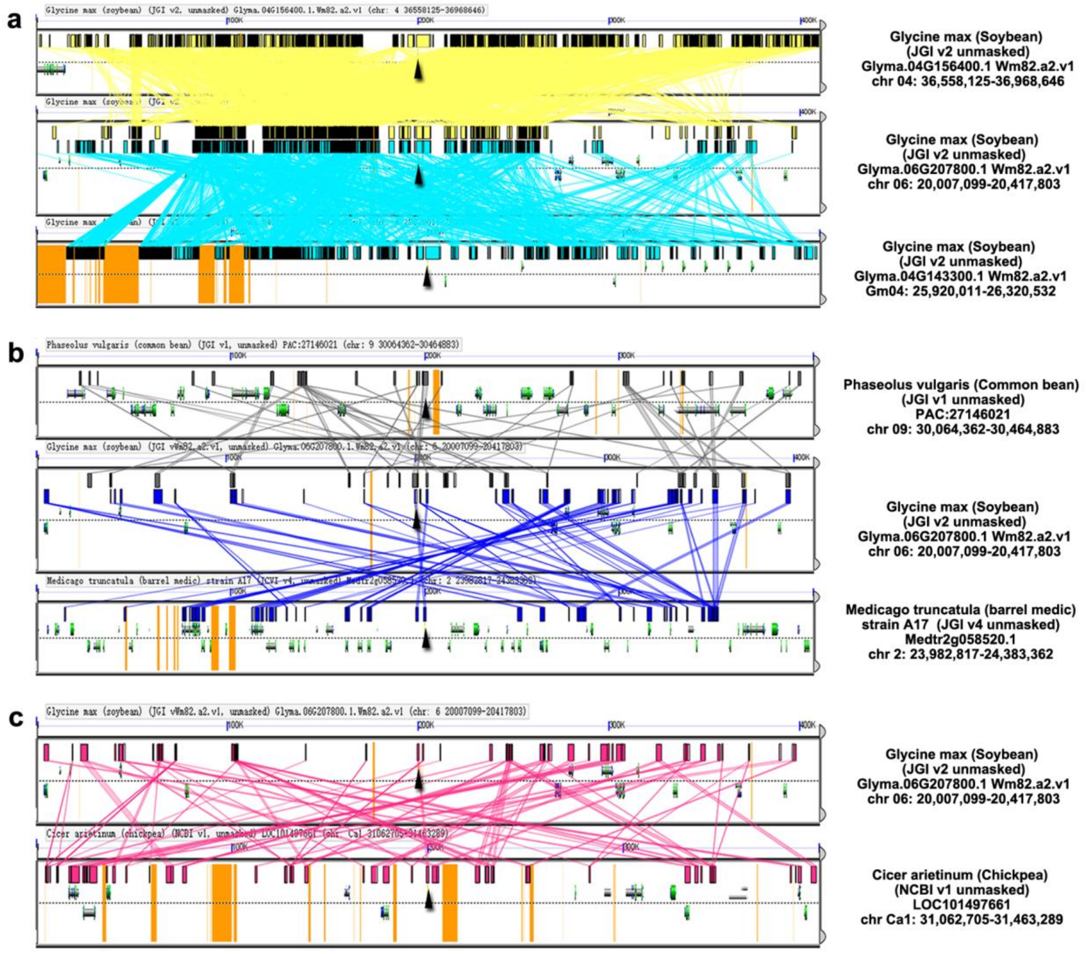

Supplementary Figure S2 Microsynteny between *E1* and its homologs from legumes.

(a) Microsynteny between the regions of *E1* and *E1La/E1Lb*. (**b)** Microsynteny between the regions of *E1* and *PvE1L/MtE1L*. (**c)** Microsynteny between the regions of *E1* and *CaE1L*. Flanking 200 kb sequences at either sides of the gene was retrieved, Repeat sequences were unmasked. Alignments were performed using BlastZ. *E1* family genes in genome are marked in black triangles. The unassembled regions in genome are marked in orange. Gene models are drawn as gray block; CDS is drawn as green arrow on the top of gray genes and blue mRNA. Colored wedges connect the conserved regions.

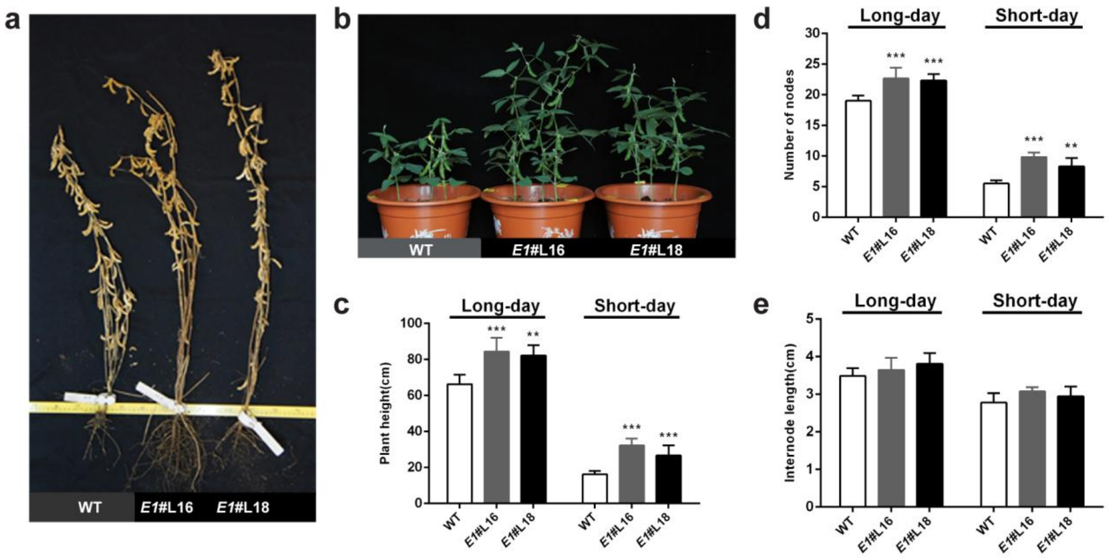

Supplementary Figure S3 Overexpression of *E1* in soybean altered plant growth.

(a–e) Differences in growth habit between WT-DN and *E1* overexpression lines under LD **(a)** and SD **(b)** conditions. Herbicide-resistant T3 plants of the transgenic lines were grown for growth habit investigation. Growth habit was measured when harvest and plants reach the maximum under LD and SD conditions, respectively. Values represent the average of six replicates +s.d. Statistical significance was determined using Dunnett's post hoc test after a significant one-way ANOVA. **, and *** indicate significant differences between transgenic lines and WT-DN plants at $P < 0.01$, and $P < 0.001$, respectively.

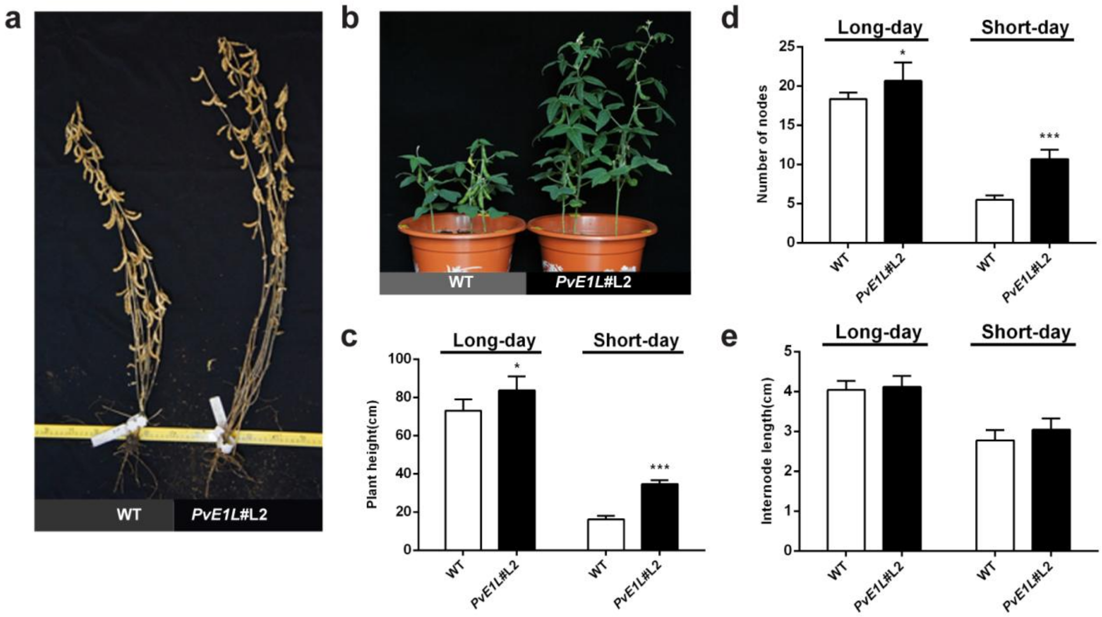

Supplementary Figure S4 Ectopic overexpression of *PvE1L* in soybean altered plant growth.

(a–e) Differences in growth habit between WT-DN and *PvE1L* overexpression lines under LD **(a)** and SD **(b)** conditions. Herbicide-resistant T3 plants of *PvE1L*#L2 were grown for growth habit investigation. Growth habit was measured when harvest and plants reach the maximum under LD and SD conditions, respectively. Values represent the average of six replicates +s.d. P-value was determined using two-tailed Students' *t*-test. *, **, and *** indicate significant differences between transgenic lines and WT-DN plants at P < 0.05, P < 0.01, and P < 0.001, respectively.

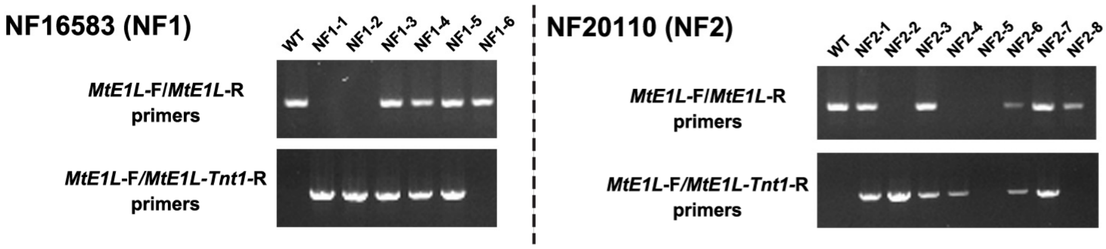

Supplementary Figure S5 Verification of *Tnt1* insertions in *Medicago* mutants.

Six plants of NF16583 (NF1) and seven plants of NF20110 (NF2), were verified using PCR. From the banding pattern, NF1-1, NF1-2, NF2-2 and NF2-4 were homozygous at *Tnt1* insertion loci in *MtE1L*. Wild-type (WT) R108 was also included as the control.

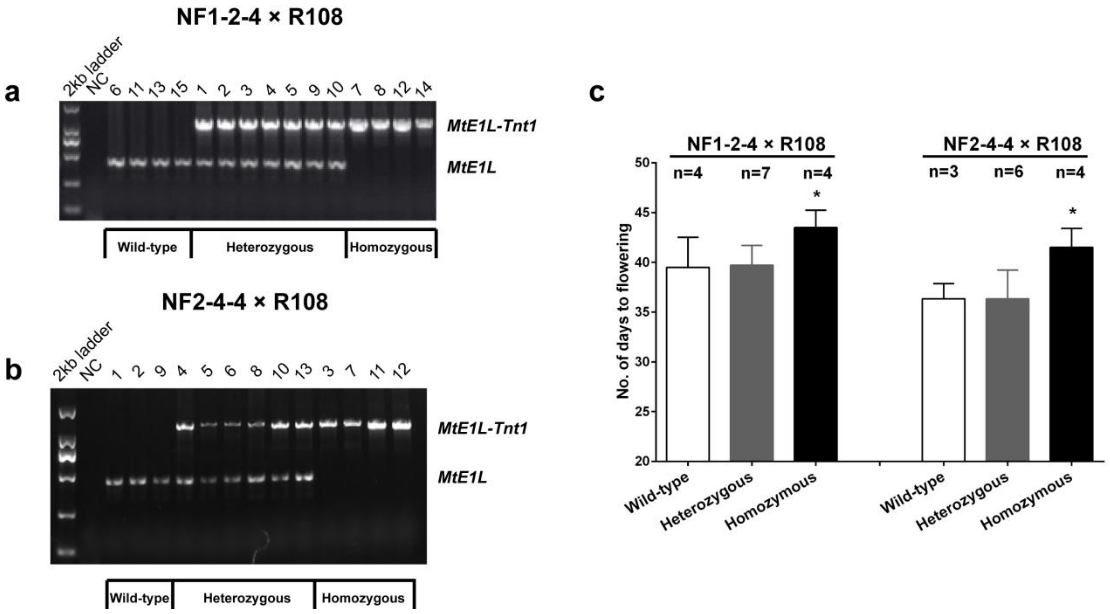

Supplementary Figure S6 Segregation analysis showing mutations at *MtE1L* were significantly associated with flowering time.

(a) Fifteen F2 plants derived from NF1-2-4 × R108 were verified using PCR. (**b)** Thirteen F2 plants derived from NF2-4-4 × R108 were verified using PCR. NC, Negative Control. (**c)** Variation of flowering time among different genotypes at mutation site. Statistical significance was determined using univariate ANOVA. * indicating difference in flowering time between homozygous mutants and WT-Mt plants reached statistical significance ($P < 0.05$).

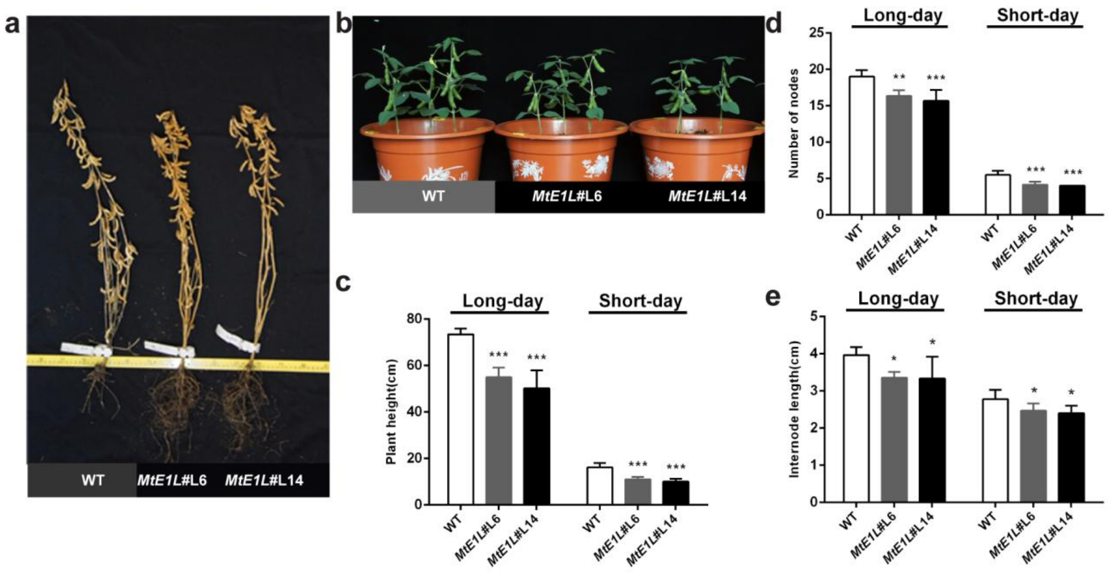

Supplementary Figure S7 Ectopic overexpression of *MtE1L* in soybean altered plant growth.

(a–e) Differences in growth habit between WT-DN and *MtE1L* overexpression lines under LD **(a)** and SD **(b)** conditions. Herbicide-resistant T3 plants of the transgenic lines were grown for growth habit investigation. Growth habit was measured when harvest and plants reach the maximum under LD and SD conditions, respectively. Values represent the average of six replicates +s.d. Statistical significance was determined using Dunnett's post hoc test after a significant one-way ANOVA. *, **, and *** indicate significant differences between transgenic lines and WT-DN plants at $P < 0.05$, $P < 0.01$, and $P < 0.001$, respectively.

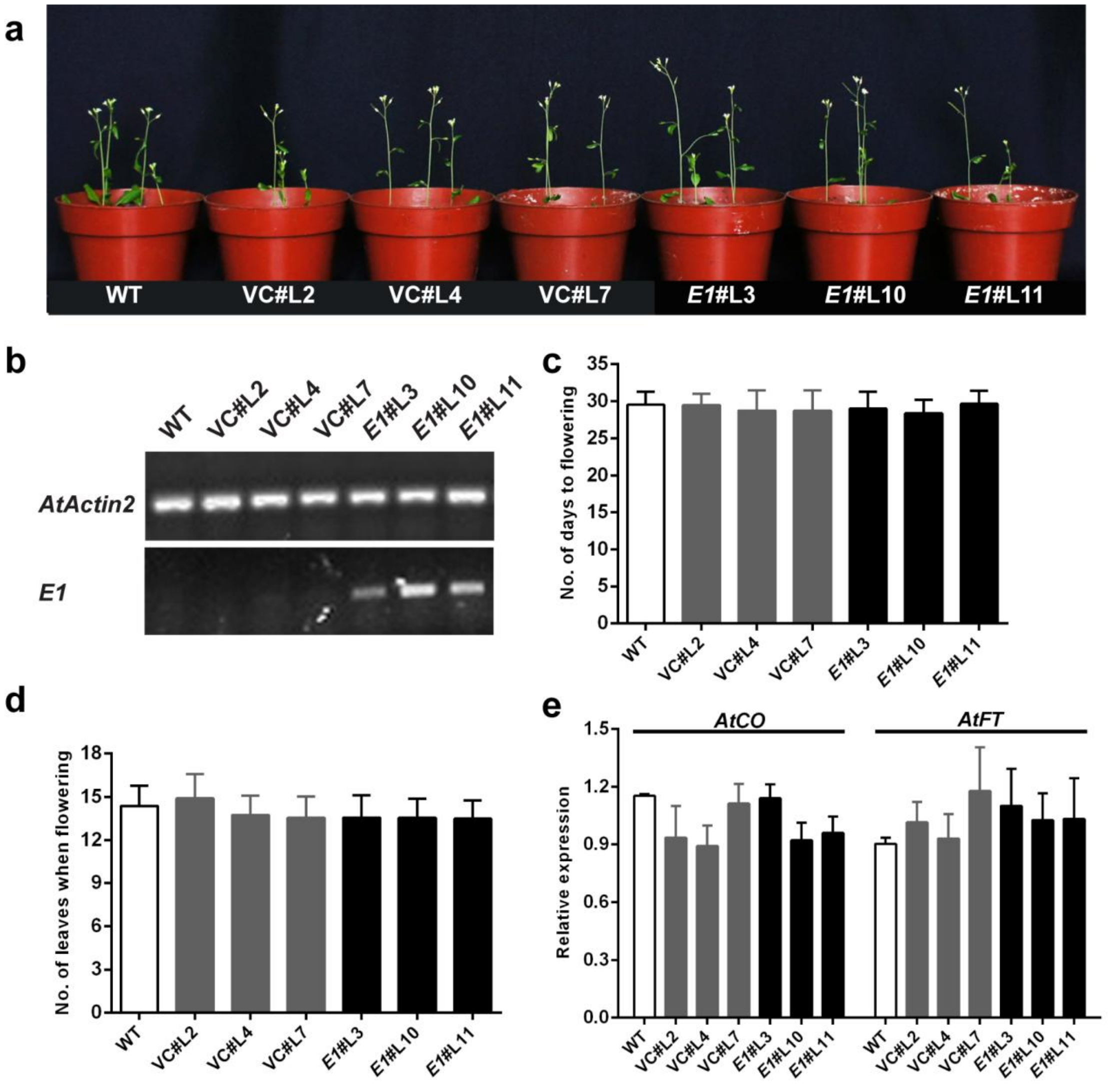

Supplementary Figure S8 Ectopic overexpression of *E1* in *Arabidopsis* led to no noticeable phenotypic changes in flowering time and on expression of flowering related genes.

(a) Flowering phenotype between WT-At, three vector-only (VC) transgenic lines (VC#L2, VC#L4 and VC#L7) and three *E1* transgenic lines (*E1*#L3, *E1*#L10 and *E1*#L11) under LD condition. (**b)** Semi-quantitative RT-PCR analysis of *E1* in wild-type *Arabidopsis Col-0* (WT-At) and all transgenic lines under LD condition. (**c)** Number of days to flowering in WT-At and all transgenic lines. As shown in (**a)** and **(c)**, there was no significant difference in flowering time between WT-At and transgenic lines, and their flowering time varied from 28.4 to 29.5 days under LD condition. **(d)** Number of rosette leaves at flowering in WT-At and all transgenic lines. T3 homozygous lines were grown for phenotype investigation. Values represent the

average of approximately 40 replications +s.d. Similar results were observed in two separate experiments. Statistical significance was determined using Dunnett’s post hoc test after a significant one-way ANOVA. **(e)** The expression of *AtCO* and *AtFT* in the fully expanded leaves of WT-At and transgenic lines under LD condition. Relative expression levels were analyzed by qRT-PCR and normalized to *AtActin2*. Values represent the average of three biological replicates +s.d.

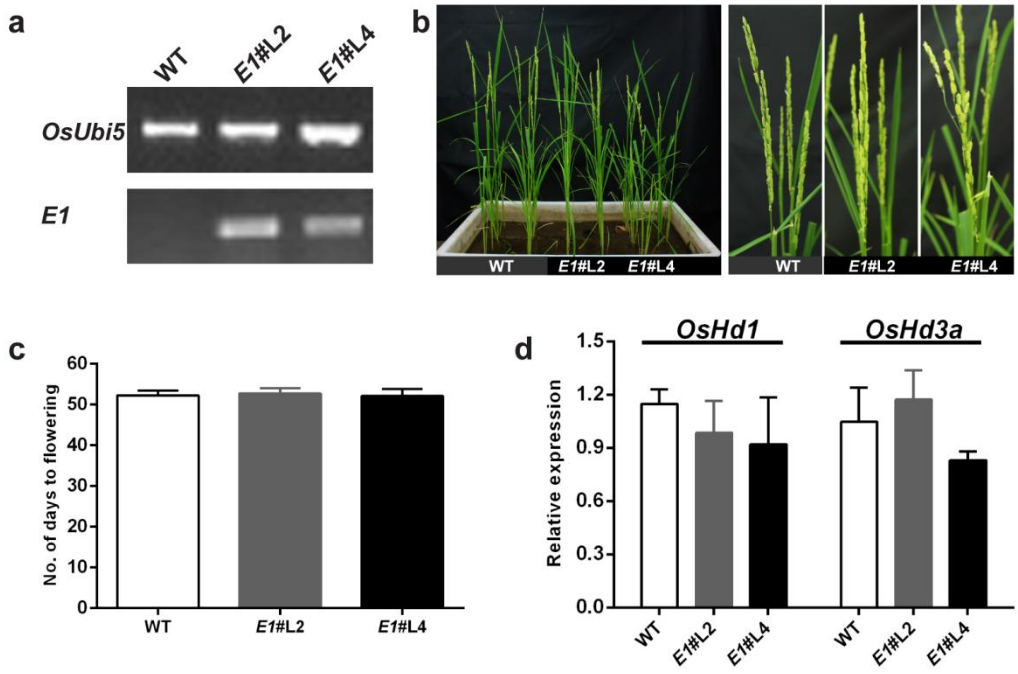

Supplementary Figure S9 Ectopic expression of *E1* in rice had no noticeable effect on heading time under SD condition and on expression of flowering related genes.

(a) Semi-quantitative RT-PCR analysis of expression levels of *E1* in two *E1* overexpression lines. (**b**) The photograph shows heading phenotype between wild-type rice cultivar Longjing 11 (WT-Os) and two *E1* overexpression lines (*E1*#L2 and *E1*#L4) under SD condition. There was no significant difference in flowering time between WT-Os and transgenic lines, and their flowering time varied from 52.1 to 52.8 days under SD condition. (**c**) Number of days to heading in WT-Os and two *E1* overxpression lines. T2 overexpression lines were grown for phenotype investigation, and heading time was recorded at a panicle was 1~2 cm long. Values represent the average of eight replications +s.d. Similar results were observed in two separate experiments. Statistical significance was determined using Dunnett’s post hoc test

after a significant one-way ANOVA. (**d**) The expression of *OsHd1* and *OsHd3a* in the fully expanded leaves of WT-Os and transgenic lines under SD condition. Relative expression levels were analyzed by qRT-PCR and normalized to *OsUbi5*. Values represent the average of three biological replicates +s.d.

Supplementary Table S1. Substitution rate and date of divergence among *E1* homologs in legumes.

Gene 1	Gene 2	Ka	Ks	Ka/Ks	Estimated time (Mya)
E1	*E1La*	0.0225	0.1077	0.2086	8.8
E1	*E1Lb*	0.0225	0.1294	0.1736	10.6
E1	*PvE1L*	0.0302	0.1723	0.1752	14.1
E1	*MtE1L*	0.1473	0.7668	0.1921	62.9
E1	*LjE1L*	0.1114	0.7418	0.1501	60.8
E1	*CcE1L1*	0.0534	0.3454	0.1545	28.3
E1	*CcE1L2*	0.0748	0.3434	0.2179	28.1
E1	*CaE1L*	0.1359	1.0795	0.1259	88.5
E1La	*E1Lb*	0.0148	0.0373	0.3969	3.1
E1La	*PvE1L*	0.0275	0.2062	0.1331	16.9
E1La	*MtE1L*	0.1474	0.7898	0.1866	64.7
E1La	*LjE1L*	0.1076	0.709	0.1517	58.1
E1La	*CcE1L1*	0.0609	0.3579	0.1702	29.3
E1La	*CcE1L2*	0.0825	0.3559	0.2318	29.2
E1La	*CaE1L*	0.1263	1.0679	0.1183	87.5
E1Lb	*PvE1L*	0.0274	0.2187	0.1254	17.9
E1Lb	*MtE1L*	0.1473	0.8763	0.1681	71.8
E1Lb	*LjE1L*	0.1075	0.6653	0.1616	54.5
E1Lb	*CcE1L1*	0.0556	0.3893	0.1429	31.9
E1Lb	*CcE1L2*	0.0824	0.387	0.213	31.7
E1Lb	*CaE1L*	0.1262	1.154	0.1094	94.6
PvE1L	*MtE1L*	0.1522	0.8465	0.1798	69.4
PvE1L	*LjE1L*	0.1036	0.8459	0.1225	69.3
PvE1L	*CcE1L1*	0.0584	0.2983	0.1958	24.5
PvE1L	*CcE1L2*	0.0854	0.2833	0.3015	23.2
PvE1L	*CaE1L*	0.128	1.1081	0.1155	90.8
MtE1L	*LjE1L*	0.1523	0.7256	0.21	59.5
MtE1L	*CcE1L1*	0.1665	0.8285	0.201	67.9
MtE1L	*CcE1L2*	0.179	0.795	0.2252	65.2
MtE1L	*CaE1L*	0.0679	0.612	0.111	50.2
LjE1L	*CcE1L1*	0.1387	0.7958	0.1743	65.2
LjE1L	*CcE1L2*	0.1452	0.7409	0.1959	60.7
LjE1L	*CaE1L*	0.1267	0.9743	0.13	79.9
CcE1L1	*CcE1L2*	0.0448	0.0476	0.9428	3.9
CcE1L1	*CaE1L*	0.1457	1.0769	0.1353	88.3
CcE1L2	*CaE1L*	0.1617	1.0918	0.1481	89.5

Supplementary Table S2. Herbicide resistance of transgenic soybean lines.

	**Herbicide (Glufosinate) testing**			
**Transgenic lines**	**T0 generation**	**T1 generation**		
		**Total**	**Resistant**	**Non-resistant**
E1#L16	Resistant	15	12	3
E1#L18	Resistant	11	9	2
PvE1L#L2	Resistant	13	10	3
MtE1L#L6	Resistant	12	9	3
MtE1L#L14	Resistant	10	8	2

Supplementary Table S3. List of primers used in this study.

primer ID	primer sequence (5'-3')	Gene name	Species	Application
PvE1L-F1	*TCTGC**TCTAGA**ATGAGCAACCCTGGAG*	*PvE1L*	Pv	**Gene cloning**
PvE1L-F1	*AAGC**GAGCTC**TTAATTCTCTGGCATAGC*			
MtE1L-F1	*ATCAGC**TCTAGA**ATGAACAATATTCACCTTCGA*	*MtE1L*	Mt	
MtE1L-R1	*AGC**GAGCTC**TTACGGCGGTATAGCC*			
E1-F	*TCAGATGAAAGGGAGCAGTGTCAAAAGAAGT*	*E1*	Gm/At/Os	**RT-PCR**
E1-R	*TCCGATCTCATCACCTTTCC*			
PvE1L-F2	*CCACCATTTGTGAAGCC*	*PvE1L*	Gm	
PvE1L-R2	*TCCGATCTCATCACCTTTC*			
MtE1L-F2	*AGAGTGTAATTGGGAGTGT*	*MtE1L*	Gm	
MtE1L-R2	*TGTCGTTGTTTTGGTTG*			
MSC27-F	*GGAGGTTGAGGGAAAGTGG*	*MSC27*	Mt	
MSC27-R	*CACCAACAAAGAATTGAAGG*			
TUA5-F	*TGCCACCATCAAGACTAAGAGG*	*TUA5*	Gm	
TUA5-R	*ACCACCAGGAACAACAGAAGG*			
MtE1L-F	*AGAGTGTAATTGGGAGTGT*	*MtE1L*	Mt	**Genotyping**
MtE1L-R	*TGTCGTTGTTTTGGTTG*			
MtE1L-Tnt1-F	*AGAGTGTAATTGGGAGTGT*	*MtE1L-Tnt1*	Mt	
MtE1L-Tnt1-R	*TGTAGGTCGGAGGTAAA*			
q*E1*-F	*CAAAAATGAAGAGGAGATGAACAATAA*	*E1*	Gm	**qRT-PCR**
q*E1*-R	*AACTCTAACTGGGGTCCCTTCAG*			
q*PvE1L*-F	*ACCCTGGAGATGAAAAGGAGC*	*PvE1L*	Gm	
q*PvE1L*-R	*CCTCTTCACTTTGGTTGCTGC*			
q*MtE1L*-F	*CCAAAACAACGACAACAAGGG*	*MtE1L*	Gm	
q*MtE1L*-R	*GTCATCAACATCCAACATAGGTAAAAT*			
q*GmFT2a*-F	*ATCCCGATGCACCTAGCCCA*	*GmFT2a*	Gm	

primer ID	primer sequence (5'-3')	Gene name	Species	Application
q*GmFT2a*-R	*ACACCAAACGATGAATCCCCA*			
q*GmFT5a*-F	*AGCCCGAACCCTTCAGTAGGGA*	*GmFT5a*	Gm	
q*GmFT5a*-R	*GGTGATGACAGTGTCTCTGCCCA*			
q*GmFT4*-F	*TTGGATCCCTTCACGAGTTC*	*GmFT4*	Gm	
q*GmFT4*-R	*TCCCTAGGTCATTTCCACGA*			
TUA5-F	*TGCCACCATCAAGACTAAGAGG*	*TUA5*	Gm	
TUA5-R	*ACCACCAGGAACAACAGAAGG*			
q*AtActin2*-F	*GCTGAGAGATTCAGATGCCCA*	*AtActin2*	At	
q*AtActin2*-R	*GTGGATTCCAGCAGCTTCCAT*			
q*AtCO*-F	*GGTGATAAGGATGCCAAGGAG*	*AtCO*	At	
q*AtCO*-R	*GGAGCCATATTTGATATTGAACTG*			
q*AtFT*-F	*TCAATCACTCTAAAGGTTACTTATGGC*	*AtFT*	At	
q*AtFT*-R	*TCTCGGAGGTGAGGGTTGCT*			
q*OsUbi5*-F	*ACCACTTCGACCGCCACTACT*	*OsUbi5*	Os	
q*OsUbi5*-R	*ACGCCTAAGCCTGCTGGTT*			
q*OsHd1*-F	*AACCAAGATCGGCAGTATGG*	*OsHd1*	Os	
q*OsHd1*-R	*GATTGATTGCTCCAGCAGGT*			
q*OsHd3a*-F	*GCTCACTATCATCATCCAGCATG*	*OsHd3a*	Os	
q*OsHd3a*-R	*CCTTGCTCAGCTATTTAATTGCATAA*			

Note: Gm: *Glycine max*; Pv: *Phaseolus vulgaris*; Mt: *Medicago trunctula*; At: *Arabidopsis thaliana*; Os: *Oryza sativa*. The recognition sites for *Xba* I and *Sac* I are colored in orange and blue, respectively.
